# Coronavirus vaccine development: from SARS and MERS to COVID-19

**DOI:** 10.1186/s12929-020-00695-2

**Published:** 2020-12-20

**Authors:** Yen-Der Li, Wei-Yu Chi, Jun-Han Su, Louise Ferrall, Chien-Fu Hung, T.-C. Wu

**Affiliations:** 1 Department of Molecular and Cellular Biology, Harvard University, Cambridge, MA USA; 2 Department of Pathology, School of Medicine, Johns Hopkins University, Baltimore, MD USA; 3 Johns Hopkins School of Medicine, 1550 Orleans St, CRB II – Room 309, Baltimore, MD 21287 USA

**Keywords:** SARS-CoV-2, Coronaviruses, Vaccine, Vaccine development

## Abstract

Severe Acute Respiratory Syndrome Coronavirus 2 (SARS-CoV-2) is a new type of coronavirus that causes the Coronavirus Disease 2019 (COVID-19), which has been the most challenging pandemic in this century. Considering its high mortality and rapid spread, an effective vaccine is urgently needed to control this pandemic. As a result, the academia, industry, and government sectors are working tightly together to develop and test a variety of vaccines at an unprecedented pace. In this review, we outline the essential coronavirus biological characteristics that are important for vaccine design. In addition, we summarize key takeaways from previous vaccination studies of Severe Acute Respiratory Syndrome Coronavirus (SARS-CoV) and Middle East Respiratory Syndrome Coronavirus (MERS-CoV), highlighting the pros and cons of each immunization strategy. Finally, based on these prior vaccination experiences, we discuss recent progress and potential challenges of COVID-19 vaccine development.

## Introduction

Coronaviruses (CoVs) are a group of related viruses that can cause respiratory tract infection in humans ranging from mild symptoms to lethal outcomes. Until now, there are seven genera of CoVs that are known to infect humans [1]. Four of these genera, including Human Coronavirus 229E (HCoV-229E), Human Coronavirus OC43 (HCoV-OC43), Human Coronavirus NL63 (HCoV-NL63), and Human Coronavirus HKU1 (HCoV-HKU1), only cause relatively mild and self-limiting respiratory symptoms [2]. Alternatively, the other three CoVs, Severe Acute Respiratory Syndrome Coronavirus (SARS-CoV), Middle East Respiratory Syndrome Coronavirus (MERS-CoV), and Severe Acute Respiratory Syndrome Coronavirus 2 (SARS-CoV-2), are highly pathogenic and can lead to severe respiratory diseases and fatal outcome in infected patients. The first lethal coronavirus SARS-CoV emerged in 2002 in Guangdong Province, China. During the 2002–2004 outbreak, SARS-CoV had infected 8,098 people and resulted in 774 SARS-associated deaths (~ 10% mortality rate) across 29 countries before it disappeared [3]. In 2012, MERS-CoV emerged in Saudi Arabia. It caused two outbreaks in South Korea in 2015 and in Saudi Arabia in 2018, and still has ongoing reports of sporadic cases nowadays. As of January 2020, there are 2,519 confirmed MERS cases and 866 deaths (~ 35% mortality rate) across 27 countries [4]. In December 2019, a new type of CoV that can cause severe respiratory illness emerged in Wuhan, China. The World Health Organization named this novel virus SARS-CoV-2 and the disease COVID-19, or Coronavirus Disease 2019. The clinical manifestation of COVID-19 can vary from asymptomatic and mild flu-like symptoms to acute respiratory distress syndrome and death. Long-term pulmonary, cardiological, and neurological complications have also been reported in COVID-19 cases [5]. Compared with SARS-CoV and MERS-CoV, SARS-CoV-2 is highly contagious with an estimated reproductive number of 2.2 (one existing COVID-19 case can cause an average of 2.2 new infections) [6]. In addition, its ability to spread through asymptomatic patients has posed a great challenge to containment measures [7]. By October 2020, SARS-CoV-2 has infected more than 43 million individuals and resulted in about 1.15 million deaths (~ 3% mortality rate) in 235 countries, areas or territories worldwide [8]. Needless to say, COVID-19 has become the most serious public health crisis of our generation and has a profound impact on the global economy and geopolitics. Although our understanding of pathogenic CoVs has been steadily accumulating for about two decades, no effective vaccine has yet been approved for the prevention of human CoV infection. Considering the rapid spread and high mortality of COVID-19, an effective vaccine is urgently needed to control this pandemic. In this review, we summarize relevant CoV biology, SARS and MERS immunization strategies, and recent efforts of COVID-19 vaccine development. We hope this review can provide essential knowledge for any researcher who is interested in COVID-19 vaccine development.

## Coronavirus biology and its implication on vaccine development

**Fig. 1:**
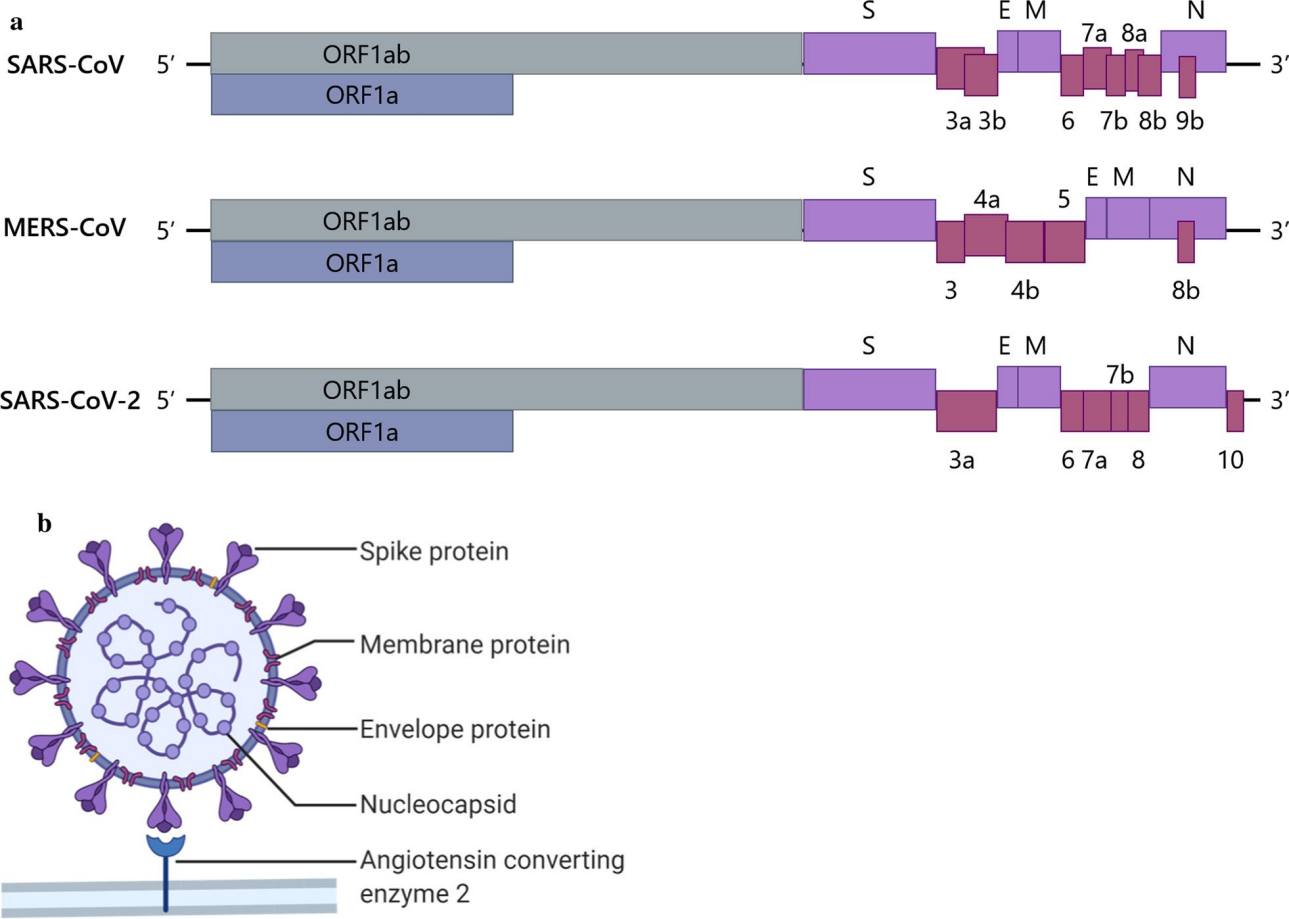
The genome and virion structure of coronaviruses (CoVs). **a** The genome structure of SARS-CoV, MERS-CoV, and SARS-CoV-2 [12–14]. The 5′-terminus of the CoV genome contains two overlapping open reading frames (ORFs): ORF 1a and ORF 1b, spanning two-thirds of the genome length. ORF 1a and ORF 1ab can be translated into two polyproteins (pp), pp1a and pp1ab, which are further cleaved into 16 non-structural proteins (Nsps). The 3′-terminus of the CoV genome encodes four major structural proteins in the order of spike (S), envelope (E), membrane (M), and nucleocapsid (N) proteins. Genus-specific accessory proteins are also encoded at the 3′-terminus of the CoV genome. **b** The virion structure of SARS-CoV-2 [16]. The spike (S), envelope (E), membrane (M) proteins form the envelope of the CoV, and the nucleocapsid (N) proteins form the capsid to pack the genomic RNA. The spike protein binds to angiotensin converting enzyme 2 (ACE2) on the cell membrane, which allows the virus to enter the cell. (Created with BioRender.com.)

Coronaviruses, whose name derives from their characteristic crown-like appearance under the electron microscope, are enveloped RNA viruses with a diameter of approximately 80–160 nm [9, 10]. The genome of CoVs is a ~ 30 kb single-stranded positive-sense RNA molecule, which is the largest genome of all known RNA viruses [9–11]. The 5′-terminus of the CoV genome contains two overlapping open reading frames (ORFs): ORF 1a and ORF 1b, spanning two-thirds of the genome length (Fig. 1a) [9–11]. ORF 1a and ORF 1ab can be translated into two polyproteins (pp), pp1a and pp1ab, which are further cleaved into 16 non-structural proteins (Nsps) involved in viral genome replication and subgenomic mRNA synthesis [9–11]. The 3′-terminus of the CoV genome encodes four major structural proteins in the order of spike (S), envelope (E), membrane (M), and nucleocapsid (N) proteins (Fig. 1a) [9–11]. S, E, M protein forms the envelope of the CoV, while N protein forms the capsid to pack genomic RNA (Fig. 1b) [9–11]. The 3′-terminus of the genome also encodes multiple accessory proteins, which are usually genus-specific and can help CoV evade the immune system or increase virulence [9–11]. For instance, SARS-CoV contains accessory protein ORF 3a, 3b, 6, 7a, 7b, 8a, 8b and 9b, MERS-CoV contains ORF 3, 4a, 4b, 5, 8b, and SARS-CoV-2 contains ORF 3a, 6, 7a, 7b, 8, 10 (Fig. 1a) [12–14].

**Fig. 2:**
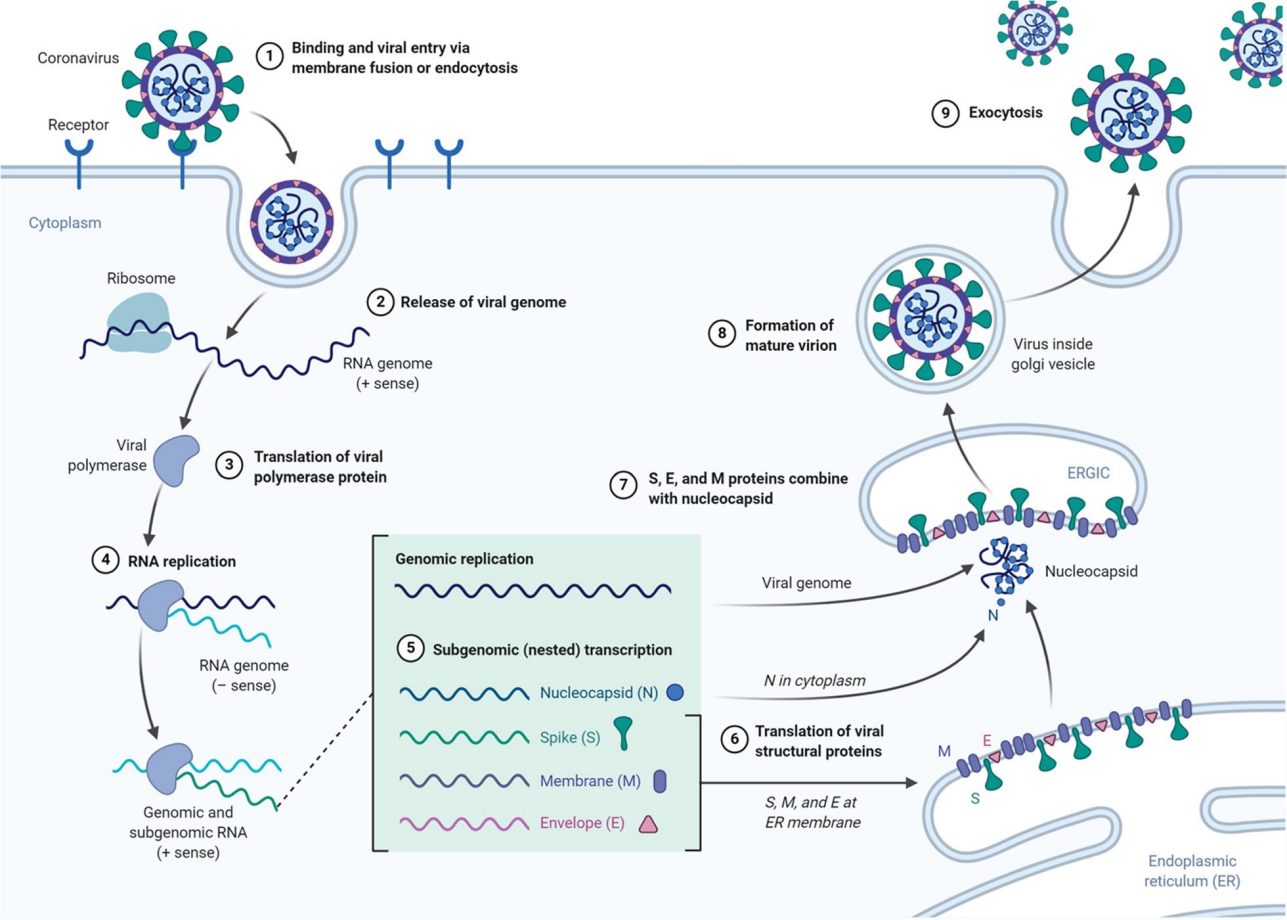
The life cycle of SARS-CoV-2 [9, 10, 15]. Upon binding to the membrane receptor ACE2, SARS-CoV-2 virion enters the host cell and releases its plus-strand RNA genome. The plus-strand RNA translates pp1a and pp1ab, which are further cleaved into multiple non-structural proteins (Nsps) including an RNA-dependent RNA polymerase (Nsp12). The RNA-dependent RNA polymerase transcribes a negative-strand genomic RNA, and then uses this negative-strand genomic RNA as template to generate more plus-strand genomic RNA (genomic replication) and many different subgenomic RNAs (subgenomic transcription). The subgenomic RNAs are further translated into major structural proteins (N, S, M, E), which will assemble with plus-strand genomic RNA to form a mature virion in lumen of the ER. Finally, the whole virus leaves the cell through exocytosis. (Reprinted from “Coronavirus Replication Cycle”, by BioRender.com (2020). Retrieved from https://app.biorender.com/biorender-templates)

Many viral proteins are essential for the life cycle of CoVs. For entering target cells, S protein first binds to cellular receptors through its receptor-binding domain (RBD), and the receptor-virus complex is subsequently translocated to endosomes (Fig. 2) [15]. Both SARS-CoV and SARS-CoV-2 S proteins bind to angiotensin-converting enzyme 2 (ACE2), while the S protein of MERS-CoV uses dipeptidyl peptidase-4 (DPP4) as its cellular receptor (Fig. 1b) [16]. At the endosome, S protein is further cleaved into S1 (RBD-containing) and S2 (non-RBD-containing) subunits, and the S2 subunit mediates fusion between the viral envelope and the host cell membrane [15]. After entering the cell, several Nsps, particularly RNA‐dependent RNA polymerase (Nsp12) and helicase (Nsp13), mediate the replication of the CoV genome and the transcription of CoV mRNA [17]. The CoV mRNA is further translated into different nonstructural and structural proteins. The N proteins bind to CoV genomic RNA to form viral nucleocapsids, and S, E, M proteins form the envelope of CoV [15]. After assembly, viral particles bud through an endoplasmic reticulum (ER)-Golgi pathway and exit the cells by exocytosis (Fig. 2) [15].

The S protein is particularly important for virus-cell receptor binding and virus-cell membrane fusion, suggesting that it can be an effective target for CoV vaccine design [15]. In fact, studies have shown that antibodies generated against the S protein are long-lasting and immunodominant in recovered SARS patients [18, 19]. In addition, several studies have demonstrated that the anti-S antibody can neutralize SARS-CoV and MERS-CoV and provides protective effects in animals and humans [20–22]. Moreover, many S protein-based vaccines against SARS-CoV and MERS-CoV have been shown to elicit potent immune responses and protective effects in preclinical models [23–27]. These results corroborate that CoV S protein serves as an ideal vaccine target to induce neutralizing antibodies and protective immunity. Besides S protein, other structural proteins have also been tested as vaccine targets. N protein-based vaccines usually cannot induce neutralizing antibodies, likely due to the fact that N protein is not displayed on the CoV surface [16]. However, N protein has the advantage of being more conserved across CoV species than S protein, making it a potential target for a T-cell inducing, universal CoV vaccine [16]. One recent study has shown that a viral vector vaccine expressing N protein can induce CD4+ T cell-dependent protection against SARS-CoV and MERS-CoV, suggesting the feasibility of N protein-based T-cell inducing CoV vaccines [28]. M protein-based vaccines, on the other hand, can induce a high titer of antibody response in immunized animals [29]. However, no neutralization antibody or protective immunity data of M protein-based vaccines in preclinical models have been demonstrated. Finally, very few CoV E protein-based immunization studies have been reported so far, and none of the studies demonstrated induction of neutralizing antibodies or protective immunity [30].

There are also immunopathological complications associated with the SARS-CoV and MERS-CoV vaccines that require addressing and further optimization. One adverse effect is the induction of antibody-dependent enhancement (ADE) effect, which is usually caused by vaccine-induced suboptimal antibodies that facilitates viral entry into host cells [11, 31]. A study found that SARS-CoV vaccine based on full-length S protein enhances SARS-CoV infection of human cell lines in vitro [32]. Additionally, two studies have also shown that anti-S protein serum results in increased viral infectivity of SARS-CoV [33, 34]. These results raise safety concerns for S protein-based SARS-CoV and MERS-CoV vaccines. One potential strategy to overcome the ADE problem is to design vaccines that only contain major neutralizing epitopes, such as the S1 subunit or the RBD domain of the S protein. This strategy can decrease the induction of non-neutralizing antibodies by CoV vaccines and therefore reduce the ADE effect. Another potential adverse effect is vaccine-induced eosinophilic immunopathology, which is an unwanted Th2-skewed immune response elicited by vaccination [11, 35]. At least two studies have reported that whole inactivated virus vaccine of SARS-CoV induces eosinophilic proinflammatory pulmonary response after mice challenged with SARS-CoV [36, 37]. In addition, one study also reported that immunization with SARS-CoV virus-like particle (VLP) vaccine leads to eosinophilic immunopathology in the lung after viral challenge [37]. In order to prevent this Th2-type immunopathology, a few studies have worked on adjuvant optimization. They found that appropriate adjuvants, such as Toll-like receptor agonist and delta-inulin polysaccharide, can increase serum neutralizing antibody titers and reduce lung eosinophilic immunopathology [38, 39]. Their results provide a promising strategy to deal with Th2-skewed immune response induced by some CoV vaccines.

## Previous progress of SARS-CoV and MERS-CoV immunization strategies

Various forms of vaccines targeting SARS-CoV and MERS-CoV have been developed and tested in preclinical models. However, only a few of them entered clinical trials and none of them have been FDA approved. These approaches include protein subunit vaccines, virus-like particle vaccines, DNA vaccines, viral vector vaccines, whole-inactivated vaccines and live-attenuated vaccines. The following sections outline the principles of various forms of SARS-CoV and MERS-CoV vaccine development (Table 1), and the latest results from both preclinical studies and clinical trials (Table 2).

**Table 1:** Advantages and disadvantages of different vaccine platforms

Vaccine platform	Advantages	Disadvantages	Clinically approved examples
Whole inactivated virus vaccine	Stronger immune response; Safer than live attenuated virus	Potential epitope alteration by inactivation process	Typhoid, Cholera, Hepatitis A virus, Plague, Rabies, Influenza, Polio (Salk)
Live attenuated virus vaccine	Stronger immune response; Preservation of native antigen; Mimicking natural infection	Risk of residual virulence, especially for immunocompromised people	Measles, Mumps, Polio (Sabin), Rota virus, Yellow Fever, Bacillus Calmette–Guérin (BCG), Rubella, Varicella
Viral vector vaccine	Stronger immune response; Preservation of native antigen; Mimicking natural infection	More complicated manufacturing process; Risk of genomic integration; Response dampened by pre-existing immunity against vector	Ebola virus
Subunit vaccine	Safe and well-tolerated	Lower immunogenicity; Requirement of adjuvant or conjugate to increase immunogenicity	Pertussis, Influenza, *Streptococcus pneumoniae, Haemophilus influenzae type b*
Viral-like particle vaccine	Safe and well-tolerated; mimicking native virus conformation	Lower immunogenicity; More complicated manufacturing process	Hepatitis B virus, Human Papillomavirus
DNA vaccine	Safe and well-tolerated; Stable under room temperature; Highly adaptable to new pathogen; Native antigen expression	Lower immunogenicity; Difficult administration route; Risk of genomic integration	NA
RNA vaccine	Safe and well-tolerated; Highly adaptable to new pathogen; Native antigen expression	Lower immunogenicity; Requirement of low temperature storage and transportation; Potential risk of RNA-induced interferon response	NA

**Table 2:** Clinical trials of SARS, MERS and COVID-19 vaccines

Platform	Vaccine		Group	Status	Ref
***SARS Vaccine Clinical Trials***					
Inactivated virus	Inactivated SARS-CoV vaccine (ISCV)		Sinovac	Phase I, completed	Lin et al. (2007) [110] No NCT ID
DNA vaccine	VRC-SRSDNA015-00-VP		NIAID	Phase I, completed	Martin et al. (2008) [65] NCT00099463
***MERS Vaccine Clinical Trials***					
DNA vaccine	GLS-5300 (INO-4700)		GeneOne Life Science/Inovio Pharmaceuticals/International Vaccine Institute	Phase I, completed	Modjarrad et al. (2019) [69] NCT02670187
DNA vaccine	GLS-5300 (INO-4700)		GeneOne Life Science/Inovio Pharmaceuticals/ International Vaccine Institute	Phase I/IIa, completed	NCT03721718
Viral vector vaccine	MVA-MERS-S		CTC North GmbH & Co. KG	Phase I, completed	Koch et al. (2020) [102] NCT03615911
Viral vector vaccine	MVA-MERS-S_DF1		CTC North GmbH & Co. KG	Phase Ib, not yet recruiting	NCT04119440
Viral vector vaccine	ChAdOx1 MERS		University of Oxford	Phase I, recruiting	Folegatti et al. (2020) [98] NCT03399578
Viral vector vaccine	ChAdOx1 MERS		King Abdullah International Medical Research Center/University of Oxford	Phase I, recruiting	NCT04170829
Viral vector vaccine	BVRS-GamVac-Combi		Gamaleya Research Institute of Epidemiology and Microbiology/Acellena Contract Drug Research and Development	Phase I/II, recruiting	NCT04128059
Viral vector vaccine	BVRS-GamVac		Gamaleya Research Institute of Epidemiology and Microbiology	Phase I/II, recruiting	NCT04130594

Platform	Vaccine	Vaccine type	Group	Status	Ref
***COVID-19 Vaccine Clinical Trials***					
Protein subunit	NVX-CoV2373	SARS-CoV-2 rS/Matrix-M1 Adjuvant	Novavax	Phase III	Keech et al. (2020) [132] 2020-004123-16 NCT04533399
RNA	mRNA-1273	LNP-encapsulated mRNA	Moderna/NIAID	Phase III	Jackson et al. (2020) [140] Anderson et al. (2020) [141] NCT04470427
RNA	BNT162b1 BNT162b2	LNP-mRNAs	BioNTech/Fosun Pharma/Pfizer	Phase III	Mulligan et al. (2020) [144] Sahin et al. (2020) [145] Walsh et al. (2020) [146] NCT04368728
Viral vector	AZD1222	ChAdOx1-S	University of Oxford/AstraZeneca	Phase III	Folegatti et al. (2020) [99] NCT04516746 NCT04540393 ISRCTN89951424 CTRI/2020/08/027170
Viral vector	Ad5-nCoV	Adenovirus Type 5	CanSino Biological Inc./Beijing Institute of Biotechnology	Phase III	Zhu et al. (2020) [92] Zhu et al. (2020) [93] NCT04526990 NCT04540419
Viral vector	Gam-COVID-Vac	Adeno-based (rAd26-S + rAd5-S)	Gamaleya Research Institute	Phase III	Logunov et al. (2020) [151] NCT04530396 NCT04564716
Viral vector	Ad26.COV2.S	Adeno-based	Janssen Pharmaceutical Companies	Phase III	NCT04505722
Inactivated virus	Adsorbed COVID-19 (inactivated) Vaccine	inactivated	Sinovac	Phase III	NCT04456595 NCT04582344 669/UN6.KEP/EC/2020
Inactivated virus	Inactivated SARS-CoV-2 vaccine (Vero cell)	Inactivated	Wuhan Institute of Biological Products/Sinopharm	Phase III	Xia et al. (2020) [154] ChiCTR2000034780 ChiCTR2000039000
Inactivated virus	BBIBP-CorV	Inactivated	Beijing Institute of Biological Products/Sinopharm	Phase III	Xia et al. (2020) [156] ChiCTR2000034780 NCT04560881
Protein subunit	Recombinant new coronavirus vaccine (CHO cell)	Adjuvanted recombinant RBD-Dimer	Anhui Zhifei Longcom Biopharmaceutical/Institute of Microbiology, Chinese Academy of Sciences	Phase II	NCT04466085
RNA	CVnCoV	mRNA	Curevac	Phase II	NCT04515147
Protein subunit	KBP-COVID-19	S protein RBD-based	Kentucky Bioprocessing, Inc	Phase I/II	NCT04473690
Protein subunit	SARS-CoV-2 vaccine	Adjuvanted S protein	Sanofi Pasteur/GSK	Phase I/II	NCT04537208
RNA	ARCT-021	mRNA	Arcturus/Duke-NUS	Phase I/II	NCT04480957
DNA	INO-4800	DNA plasmid with electroporation	Inovio Pharmaceuticals/International Vaccine Institute	Phase I/II	NCT04447781 NCT04336410
DNA	AG0301-COVID19	Adjuvanted DNA plasmid	Osaka University/AnGes/Takara Bio	Phase I/II	NCT04463472 NCT04527081
DNA	nCov Vaccine	DNA plasmid	Cadila Healthcare Limited	Phase I/II	CTRI/2020/07/026352
DNA	GX-19	DNA Vaccine	Genexine Consortium	Phase I/II	NCT04445389
Inactivated	BBV152A BBV152B BBV152C	Inactivated	Bharat Biotech	Phase I/II	NCT04471519 CTRI/2020/09/027674
Inactivated	Inactivated SARS-CoV-2 Vaccine	Inactivated	Institute of Medical Biology, Chinese Academy of Medical Sciences	Phase I/II	NCT04470609
Inactivated	QazCovid-in	Inactivated	Research Institute for Biological Safety Problems, Rep of Kazakhstan	Phase I/II	NCT04530357
VLP	RBD SARS-CoV-2 HBsAg VLP	RBD-HBsAg VLPs	SpyBiotech/Serum Institute of India	Phase I/II	ACTRN12620000817943
Protein subunit	SCB-2019	Adjuvanted S protein	Clover Biopharmaceuticals Inc./GSK/Dynavax	Phase I	NCT04405908
Protein subunit	COVAX-19	S protein with Advax-SM adjuvant	Vaxine Pty Ltd/Medytox	Phase I	NCT04453852
Protein subunit	SARS-CoV-2 Sclamp vaccine	Molecular clamp stabilized S protein with MF59 adjuvant	University of Queensland/CSL/Seqirus	Phase I	ACTRN12620000674932p ISRCTN51232965
Protein subunit	MVC-COV1901	S-2P protein + CpG 1018	Medigen Vaccine Biologics Corporation/NIAID/Dynavax	Phase I	NCT04487210
Protein subunit	Soberana 01	S protein RBD with Adjuvant	Instituto Finlay de Vacunas, Cuba	Phase I	IFV/COR/04
Protein subunit	EpiVacCorona	Adjuvanted peptide antigen	FBRI SRC VB VECTOR, Rospotrebnadzor, Koltsovo	Phase I	NCT04527575
Protein subunit	Recombinant SARS-CoV-2 vaccine	S protein RBD (Sf9 cells)	West China Hospital, Sichuan University	Phase I	ChiCTR2000037518
Protein subunit	IMP (CoVac-1)	Multipeptide cocktail of SARS-CoV-2 HLA-DR peptides	University Hospital Tuebingen	Phase I	NCT04546841
Protein subunit	UB-612	S1-RBD-protein	COVAXX	Phase I	NCT04545749
RNA	LNP-nCoVsaRNA	self-amplifying ribonucleic acid (saRNA) encoding S protein	Imperial College London	Phase I	ISRCTN17072692
RNA	SARS-CoV-2 mRNA vaccine	mRNA encoding S protein RBD	People's Liberation Army (PLA) Academy of Military Sciences/Walvax Biotech	Phase I	ChiCTR2000034112
Viral vector	hAd5-S-Fusion + N-ETSD vaccine	hAd5 Spike (S) + Nucleocapsid (N)	ImmunityBio, Inc. & NantKwest Inc	Phase I	NCT04591717
Viral vector	GRAd-COV2	Replication defective Simian Adenovirus (GRAd)	ReiThera/LEUKOCARE/Univercells	Phase I	NCT04528641
Viral vector	Ad5-nCoV	Ad5-based	CanSino Biological Inc/Institute of Biotechnology, Academy of Military Medical Sciences, PLA of China	Phase I	NCT04552366
Viral vector	VXA-CoV2-1	dsRNA-adjuvanted Ad5	Vaxart	Phase I	NCT04563702
Viral vector	MVA-SARS-2-S	MVA + spike protein (S)	Ludwig-Maximilians - University of Munich	Phase I	NCT04569383
Viral vector	V590	VSV + S protein	Merck Sharp & Dohme/IAVI	Phase I	NCT04569786
Viral vector	TMV-083	Measles-vector based	Institute Pasteur/Themis/Univ. of Pittsburg CVR/Merck Sharp & Dohme	Phase I	NCT04497298
Viral vector	DelNS1-2019-nCoV-RBD-OPT1	Intranasal flu-based-RBD	Beijing Wantai Biological Pharmacy/Xiamen University	Phase I	ChiCTR2000037782
Inactivated	inactivated SARS-CoV-2 Vaccine	Inactivated	Beijing Minhai Biotechnology	Phase I	ChiCTR2000038804
VLP	Recombinant Coronavirus-Like Particle COVID 19 Vaccine	CpG 1018- or AS03-adjuvanted Plant-derived VLP	Medicago Inc	Phase I	NCT04450004

## Protein subunit vaccine

Protein subunit vaccines consist of viral antigenic fragments produced by recombinant protein techniques. They are easy to produce, and relatively safe and well-tolerated compared to whole virus vaccines and viral vector vaccines. The drawback of protein subunit vaccines is their low immunogenicity. Therefore, adjuvants and fusion with immunostimulatory molecules are usually used together with subunit vaccines to overcome this challenge.

The development of SARS-CoV protein subunit vaccines was initially surrounding full-length S protein-based vaccines and then later focused on S protein RBD-based vaccines. None of the SARS-CoV protein subunit vaccines entered clinical trials, but they induced potent antibody responses and protective effects in preclinical models [23, 24, 32, 40–44]. Studies have shown that full-length S protein, extracellular domain of the S protein, and trimeric S proteins (triSpike) are all immunogenic and can elicit protection against SARS-CoV infection [23, 24, 32]. However, Kam et al. and Jamue et al. have found that triSpike vaccine can also cause Fcγ receptor II (FcγRII)-dependent SARS-CoV infection in human B cells in vitro [32, 33]. On the other hand, S protein RBD-based vaccines are able to induce high-titer neutralizing antibodies without causing obvious pathogenic effects [40–44]. This is probably because RBD-based vaccines do not contain additional non-neutralizing epitopes as full-length S protein vaccines do. One study has shown that RBD-based vaccines not only protect most of the SARS-CoV challenged mice with no detectable viral RNA in the lung, but can also induce long-lasting S-specific antibodies that can be maintained for 12 months [42]. Furthermore, RBD-based SARS-CoV vaccines have also been shown to induce RBD-specific IFN-γ producing cellular immune responses in mice [44]. As a result, SARS-CoV RBD has become the main target for SARS vaccines. Finally, SARS-CoV subunit vaccines based on S2 subunit, N and M structural proteins have also been tested [29, 45, 46]. However, no evidence has shown that they can induce neutralizing antibodies or protective effects against viral challenge.

Guided by previous SARS-CoV experiences, most proteins subunit vaccines of MERS-CoV are focused on RBD-based vaccines. RBD-based MERS-CoV vaccines generally show great immunogenicity and elicit potent neutralizing antibodies, cell-mediated immunity, and protective effect against MERS-CoV infection [25, 26]. One study from Tai et al. found that trimeric RBD protein vaccines can induce long-lasting neutralizing antibodies for 6 months [26]. Another study also from Tai et al. demonstrated that recombinant RBD proteins from different MERS-CoV strains can induce antibodies that cross-neutralize with divergent human and camel MERS-CoV strains [25]. These results indicate that MERS-CoV RBD serves as a promising vaccine target with the ability to induce long-lasting and broad-spectrum neutralizing antibodies against infection. Other than RBD-based vaccines, RBD-containing S1 subunit vaccines have also been shown to induce neutralizing antibodies and protection against MERS-CoV [47, 48]. Notably, the N-terminal domain (NTD) of the S protein binds to sialic acid and is important for MERS-CoV infection in certain cell types. Jiaming et al. showed that immunization with NTD-based vaccine also provides protection against MERS-CoV and induces potent humoral and cell-mediated immunity [49]. However, since SARS-CoV-2 NTD does not have the same sialic acid-binding function as MERS-CoV, NTD-based strategy might not be generalizable to SARS-CoV-2 vaccine development.

Apart from antigen design, several other factors also affect the efficacy of protein subunit vaccines [16]. The expression system of protein influences the quality and quantity of protein subunit vaccines. Du et al. demonstrated that SARS-CoV RBD protein expressed by mammalian 293T cells induces stronger neutralizing antibody responses than RBD expressed by insect cells and *E. coli*, which is probably due to the native conformation and post-translational modification maintained in mammalian cellular system [43]. In addition, adjuvants play an important role in enhancing the immunogenicity of protein subunit vaccines. Zhang et al. have examined multiple adjuvants (Freund’s, aluminum, Monophosphoryl lipid A, Montanide ISA51 and MF59) in conjugation with MERS-CoV RBD and found that MF59 best potentiate the protein to elicit neutralizing antibodies and protective effects [50]. Their data provide a good starting point for optimizing adjuvants for SARS-CoV-2 subunit vaccines. Moreover, the immunization route of the subunit vaccine can also affect its potency, and in combination with different antigen and adjuvants, the optimized vaccination pathway may change. For example, Li et al. demonstrated that for SARS-CoV S and S1 subunit vaccine, intramuscular (I.M.) route induces stronger antibody responses than subcutaneous (S.C.) route, while Lan et al. showed that S.C. route is preferable over I.M. injection for Freund’s and CpG-adjuvanted MERS-CoV RBD vaccine [23, 51]. Therefore, the ideal route might need to be customized for SARS-CoV-2 subunit vaccines.

## Virus-like particle vaccine

Virus-like particles (VLPs) are self-assembled viral structural proteins that mimic the conformation of native viruses but lack the viral genome. Compared with protein subunit vaccines, VLP vaccines present epitope in conformation that is more similar to the native virus, leading to better immunization responses. In addition, compared to whole virus vaccines, the production of VLP vaccines does not involve live virus or inactivation steps, which makes them safer vaccine candidates. The highly repetitive antigenic surface of VLP vaccines also help induce stronger antibody response by efficiently cross-linking B-cell surface receptors. Up to now, VLP vaccines have been commercialized for the protection against human papillomavirus (Cervarix™ and Gardasil®) and hepatitis B virus (Engerix® and Recombivax HB®) [52].

Few SARS-CoV and MERS-CoV VLP vaccines have been reported so far. For SARS-CoV, Lokugamage et al. have demonstrated that chimeric VLPs composed of SARS-CoV S protein and mouse hepatitis virus E, M and N proteins can induce neutralizing antibody responses and reduce SARS-CoV virus titer in mice lung after viral challenge [53]. In addition, another study performed by Liu et al. showed that chimeric VLPs consisting of SARS-CoV S protein and influenza virus M1 protein can induce neutralizing antibodies and provide protection against lethal challenge in mice [54]. However, one study used the same chimeric VLPs as Lokugamage et al. and showed that this VLP vaccine can lead to pulmonary immunopathology on challenge with SARS-CoV [37, 53]. Therefore, potential adverse effects of coronavirus VLP vaccines should be monitored. For MERS-CoV VLP vaccines, Wang et al. have shown that VLPs containing MERS-CoV S, E and M proteins can induce specific antibody response and Th1-mediated cellular immunity in rhesus macaques [55]. The same research group developed another chimeric VLP vaccine containing the fusion of the receptor-binding domain (RBD) of MERS-CoV S protein and the canine parvovirus (CPV) VP2 structural protein [56]. They showed that this VLP vaccine induces MERS-CoV-specific antibody response and T-cell immunity in mice [56]. These studies suggested that VLP vaccines hold the potential for clinically effective coronavirus vaccines.

## DNA vaccine

DNA vaccines contain genes encoding viral antigenic components that are expressed by plasmid vectors and delivered into cells through electroporation. Compared with other vaccine technologies, DNA vaccines offer a fast and flexible platform for vaccine development and production, making it an attractive technology to combat emerging epidemics like SARS-CoV-2. In addition, antigen production of DNA vaccines happens in the target cells, which helps recapitulate the native conformation and post-translational modification of viral antigens. However, an important drawback of DNA vaccines is their limited immunogenicity due to their inability to spread and amplify in vivo. Therefore, it is important to consider strategies that can enhance the potency of DNA vaccines, such as adding adjuvant or using a prime-boost regimen. Besides, the genomic integration of DNA vaccines into the host chromosome is another biosafety concern, which may lead to mutagenesis and oncogenesis [57]. Even though previous studies have shown that the risk of vaccine plasmid insertion into the host chromosome is pretty low, the FDA and the WHO still recommends integration studies be included as part of the safety program of DNA vaccines [58, 59].

Several DNA vaccine candidates have been reported for SARS-CoV, including the S-, M-, and N protein-based vaccines [60–64]. Although all of them can generate a certain level of antibody and cell-immune responses, only S protein-based DNA vaccine has been shown to induce protective effect against SARS-CoV infection, probably due to the indispensable role of S protein in receptor binding [60]. Yang et al. has demonstrated that immunization with DNA encoding full-length S protein, S protein lacking part of cytoplasmic domain, S protein lacking both cytoplasmic and transmembrane domains can all induce neutralizing antibodies and T-cell immune responses, as well as providing protective effect in mice [60]. This promising result leads to a following phase I clinical trial based on SARS-CoV full-length S protein DNA vaccine, which showed that the vaccine was well-tolerated in patients and can induce neutralizing antibodies and T cell responses in healthy adults [65]. Furthermore, two studies have made use of prime-boost strategy to enhance the potency of S protein-based SARS-CoV DNA vaccine. Zakhartchouk et al. reported that the combination of DNA and whole-inactivated SARS-CoV vaccines can increase the magnitude of antibody response as well as inducing a more desirable Th1-skewed immune response [66]. Woo et al. demonstrated that using DNA vaccine priming plus *E. coli* expressed recombinant S protein booster can also induce higher neutralization titers than DNA or protein subunit vaccine alone [67].

Similar to SARS-CoV, several studies on MERS-CoV DNA vaccines have demonstrated optimistic results. Muthumani et al. reported that a full-length S protein-based MERS-CoV DNA vaccine can induce potent cellular immunity and antigen-specific neutralizing antibodies in mice, macaques, and camels, and macaques vaccinated with this DNA vaccine were protected against MERS-CoV challenge without demonstrating any clinical or radiographic signs of pneumonia [68]. Building on these encouraging data, a phase I clinical trial based on this MERS-CoV DNA vaccine (GLS-5300, or INO-4700) has been completed [69]. The results showed that GLS-5300 is well tolerated with no vaccine-associated serious adverse events, and immunization with GLS-5300 induces durable immune responses in 85% of participants after two vaccinations [69]. These data support further development of the GLS-5300 vaccine. Notably, a SARS-CoV-2 DNA vaccine candidate, INO-4800, is based on the same design as GLS-5300, and this vaccine is now in phase I/II clinical trial (NCT04447781 and NCT04336410) [70]. In addition, another MERS-CoV vaccine study using full-length S protein DNA priming plus S1 protein subunit booster elicits robust serum-neutralizing activity against several MERS-CoV strains in mice and rhesus macaques [47]. Immunizing rhesus macaques with this DNA prime/protein boost vaccine confers protection against MERS-CoV-induced radiographic pneumonia, supporting this strategy as a promising approach for MERS-CoV vaccine development [47]. Aside from full-length S, S1 subunit is also a good target for MERS-CoV DNA vaccine. One study performed by Al-Amri et al. has compared the immunogenicity of full-length S-based (pS) and S1-based (pS1) MERS-CoV vaccine using the same expression vector [71]. They found that pS1 immunization elicited a balanced Th1/Th2 response and generally higher levels of all IgG isotypes compared to pS vaccination, which may be explained by the fact that the transmembrane domain-lacking S1 subunit is secreted more efficiently to the extracellular space and therefore result in greater uptake by antigen-presenting cells [71]. This study suggested that S1 might be a better target than full-length S for MERS-CoV DNA vaccine [71].

Taken together, DNA vaccines encoding full-length S or S1 protein have demonstrated encouraging results to fight against SARS-CoV and MERS-CoV. The same strategy is likely to be generalizable to SARS-CoV-2 DNA vaccine considering the biological similarity.

## Viral vector vaccine

Viral vector vaccines are recombinant viruses that encode antigens of interest in an unrelated modified virus. They deliver antigen into the cells mimicking natural infection, so they induce strong antigen-specific cellular and humoral immune responses per se, thereby obviating the need for additional adjuvants. In addition, viral vectors are able to accept large insertions in their genome, providing a flexible platform for antigen design. Despite these advantages, there are several drawbacks. The manufacturing process for viral vector vaccines is more complicated than other approaches, including the optimization of cellular systems and the exclusion of contaminants, which can greatly affect the efficiency of viral vectors [57]. Moreover, recombinant viruses carry the risk of integrating their genome into the human host, so additional biosafety assessment will be required before entering clinical trials. Finally, if the chosen viral vector can infect the general populations, the pre-existing immunity on the viral vector could dampen the induced immune response, which has been seen in adenovirus- and measle virus-based vaccines [72, 73].

Similar to DNA and protein subunit vaccines, most viral vector coronavirus vaccines target the S antigen. Numerous viral vectors have been used to develop SARS-CoV and MERS-CoV vaccines, which have been described in detail in previous review articles [74, 75]. In the following sections, we will highlight vaccines based on adenovirus, modified vaccinia virus Ankara and Venezuelan equine encephalitis virus, which are the most well studied viral vector platforms for coronavirus vaccines. We will also briefly introduce other recombinant viral platforms that are being actively developed for coronavirus vaccines.

### SARS-CoV viral vector vaccine

Adenovirus is a popular viral vector vaccine that has been tested in clinical trials for a wide variety of diseases, and several studies have also examined the efficacy of adenovirus-based SARS-CoV vaccine. The feasibility of SARS adenovirus vector vaccine was first demonstrated in two study by Gao et al. and Liu et al. [76, 77]. They showed that adenoviral vector expressing S1 fragment can induce neutralizing antibodies in monkeys and rats, respectively, but neither studies showed evidences of in vivo protection against SARS-CoV challenge [76, 77]. Later on, See et al. compared the efficacy of SARS-CoV S protein expressing adenovirus vaccine with the whole inactivated SARS-CoV vaccine [78]. They found that both vaccines induce protective effects in mice against SARS-CoV challenge, though the neutralizing antibody response is weaker in adenovirus-based vaccine than whole-inactivated virus vaccine [78]. Besides, Kobinger et al. have also tested a prime-boost regimen of S protein-expressing human adenovirus type 5 and chimpanzee derived adenoviruses in ferrets [79]. Their results showed that this vaccine leads to a substantial reduction in viral load and prevents pneumonia in ferrets after SARS-CoV challenge [79]. All these results have encouraged subsequent development of adenovirus-based MERS and COVID-19 vaccines.

Modified vaccinia virus Ankara (MVA) is another well-established vaccine platform to combat emerging infectious diseases [80]. Bisht et al. has shown that intranasal or intramuscular immunization with highly attenuated MVA containing full-length S gene induces both neutralizing antibody responses and protective immunity in mice, evident by reduced virus titer in mice lung post SARS-CoV challenge [81]. Another study performed by Chen et al. demonstrated that recombinant MVA expressing SARS-CoV S protein elicits neutralizing antibodies in mice, ferrets, and monkeys, but they didn’t show any protection experiment data in this study [82]. However, another two studies performed by Weingartl et al. and Czub et al. showed that MVA vaccine expressing SARS-CoV S protein does not provide protective effect in ferrets, and it even induces inflammatory responses and focal necrosis in the liver [83, 84]. Therefore, potential adverse effects need to be considered for MVA-based SARS-CoV S protein vaccine.

For Venezuelan equine encephalitis (VEE) virus-based SARS-CoV vaccine, Deming et al. has reported that VEE virus replicon particles (VRP) expressing S protein provides complete short- and long-term protection against homologous strain challenge in young and senescent mice [85]. To further improve the efficacy of VEE virus-based vaccine against heterologous SARS-CoV challenge, Sheahan et al. has improved the immunogenicity of VRP S protein vaccine by substituting an attenuated VEE glycoprotein with its wild-type counterpart, and their result showed that the improved VRP S protein vaccine can protect aged mice from heterologous SARS-CoV challenge [86].

Several additional viral vectors have also shown promising results for SARS-CoV vaccines. Two studies from Buchholz et al. and Bukreyev et al. have used an attenuated parainfluenza virus as vector to express SARS-CoV S protein, showing that parainfluenza-based vaccine can induce neutralizing antibody responses and protective effect against SARS-CoV challenge in hamsters and monkeys [30, 87]. Besides, attenuated vesicular stomatitis virus (VSV) have also been tested as SARS-CoV vaccine vectors by Kapadia et al. [88]. Their results showed that immunization with recombinant VSV expressing S protein can induce SARS-neutralizing antibodies and is able to protect mice from SARS-CoV infection [88].

### MERS-CoV viral vector vaccine

Several adenovirus-based MERS-CoV vaccines have been developed. Human adenovirus type 5 (Ad5) and type 41 (Ad41) expressing MERS-CoV S or S1 protein have been shown to induce neutralizing antibodies in mice [89, 90]. However, the protection effect of Ad5- and Ad41-based MERS vaccines have not been evaluated [89, 90]. Notably, Ad5-MERS-S vaccine has been used in combination with S protein nanoparticles [91]. Heterologous immunization by priming with Ad5/MERS and boosting with spike protein nanoparticles has demonstrated not only protective effect in hDPP4-transduced mice against MERS-CoV challenge, but also more balanced Th1/Th2 responses than Ad5- or nanoparticles-alone homologous prime-boost vaccines [91]. The Ad5 vector has already been applied to SARS-CoV-2 vaccine development, and promising results have been demonstrated in phase I and II clinical trials [92, 93].

Besides, chimpanzee adenovirus has been employed as viral vector with an aim to overcome the pre-existing immunity problem of human adenoviruses. A MERS-CoV S protein vaccine based on a chimpanzee adenoviral vector (ChAdOx1) was shown to induce high levels of neutralizing antibodies and cell-mediated immune in mice, and to protect hDPP4-transduced mice from lethal MERS-CoV challenge [94, 95]. In addition, ChAdOx1-MERS vaccine has also been demonstrated to reduce viral load in dromedary camels and provide protective immunity in rhesus macaques [96, 97]. Given these promising preclinical results, the ChAdOx1-MERS vaccine has entered a phase I clinical trial, and the trial result showed that ChAdOx1-MERS was safe and well tolerated at all tested doses, and a single dose was capable of inducing both humoral and cellular responses against MERS-CoV [98]. Notably, the same research team has applied ChAdOx1 platform to SARS-CoV-2 vaccine development and their product AZD1222 (or ChAdOx1-nCoV-19) is now a leading player of the COVID-19 vaccine race [99].

The vaccine of modified vaccinia virus Ankara (MVA) expressing full-length MERS-CoV S protein has been reported to induce not only virus-neutralizing antibody responses and MERS-CoV-specific CD8+ T cell response, but also provide protective effect against MERS-CoV in DPP4-transduced mice [100]. Furthermore, dromedary camels immunized with this MVA-based MERS-CoV S protein vaccine generate neutralizing antibodies and show less virus excretion after MERS-CoV infection [101]. Since camel is a major animal reservoir for MERS-CoV, this vaccine provides an opportunity to effectively control camel-to-human transmission [101]. Finally, a phase I clinical trial showed that MVA-MERS-S vaccine has a favorable safety profile, and homologous prime–boost immunization of MVA-MERS-S vaccine induces humoral and cell-mediated immune responses against MERS-CoV, which supports testing MVA-MERS-S vaccine in a larger population [102].

MERS-CoV vaccines dependent on Venezuelan equine encephalitis (VEE) virus have also been studied. Agnihothram et al. have demonstrated that VEE virus replicon particles (VRP) expressing MERS-CoV S protein can induce neutralizing antibodies in young and aged mice [103]. Another study from Zhao et al. found that VRP-based MERS N protein vaccine can induce memory CD4+ immune response and provide protective immunity against MERS-CoV in hDPP4-transduced mice [28]. Since N protein is more conserved than S protein across different coronavirus species, their approach might hold the potential to develop a universal T cell-inducing coronavirus vaccine [28].

Several other vaccine platforms have been applied for the development of MERS-CoV vaccine. Measle virus- and rabies virus-based MERS-CoV S protein vaccines have been shown to induce neutralizing antibodies and provide protective effect against MERS-CoV in hDDP4-transduced mice [104, 105]. Newcastle disease virus and vesicular stomatitis virus vectors have also been employed as S protein-expressing MERS vaccines [106, 107]. However, only in vitro neutralization data have been provided and no in vivo protection data has been demonstrated for these two vaccines [106, 107].

In summary, SARS-CoV and MERS-CoV vaccines based on many viral vectors, including adenovirus, modified vaccinia virus Ankara, Venezuelan equine encephalitis virus, parainfluenza virus, vesicular stomatitis virus, Measle virus, and rabies virus, have been shown to elicit protective immunity against viral challenges. Some of these viral vectors has already become promising candidate platforms for the development of SARS-CoV-2 vaccine.

## Whole inactivated vaccine

Whole inactivated vaccines are composed of chemically or radiationally inactivated virions. They contain a full repertoire of immunogenic components of the original virus, and compared with attenuated viruses, they carry no risk of viral reactivation if properly inactivated. Although safer than live attenuated vaccines, the immunogenic epitopes of inactivated viruses may be structurally deformed during the inactivation process, which can undermine the protection they may provide. Moreover, both SARS-CoV and MERS-CoV whole inactivated vaccines have been reported to induce eosinophil-related lung pathology [36, 37]. These disadvantages make whole inactivated vaccines a less attractive strategy for coronavirus vaccine development.

During the early development of SARS-CoV vaccines, inactivated whole virus was once a leading strategy. Studies have shown that UV- and formaldehyde-inactivated SARS-CoV can induce neutralizing antibody response, and a phase I clinical trial using β-propiolactone-inactivated SARS-CoV vaccine demonstrated that it is safe, well-tolerated, and can elicit SARS-CoV-specific neutralizing antibodies [108–110]. However, later studies found that a UV-formaldehyde doubly inactivated SARS-CoV vaccine, either unadjuvanted or alum-adjuvanted, provides incomplete protection in mice and induces eosinophilic pulmonary inflammatory response upon SARS-CoV challenge [36]. Similarly, gamma-irradiated MERS-CoV vaccine adjuvanted with alum or MF59 also induces eosinophil-related lung pathology after virus challenge, despite its ability to induce neutralizing antibodies [111]. These results have dampened the enthusiasm of whole-inactivated coronavirus vaccines. Nevertheless, recently two studies have revealed that UV-inactivated SARS-CoV adjuvanted with Toll-like receptor agonists, and formaldehyde-inactivated MERS-CoV adjuvanted with alum and unmethylated CpG, can reduce or even prevent Th2-skewed lung pathology after challenge [38, 112]. These results demonstrated that with an appropriate combination of inactivation method and adjuvants, the whole inactivated virus is still a viable option for coronavirus vaccine development.

## Live attenuated vaccine

Live attenuated vaccines are live viruses weakened by deleting or mutating the pathogenic component of the viral genome. Similar to whole inactivated vaccines, they possess nearly the full immunogenic components of the original virus. Furthermore, they preserve the native conformation of viral antigens and present antigens to the immune system as in natural infections. Therefore, live attenuated vaccines are the most immunogenic kind of vaccine and have a long history of success in controlling a variety of infectious diseases [113]. However, live attenuated vaccines also carry a higher risk than other types of vaccines, including the possibility of reversion to a virulent state and the danger of persistent infection in immunocompromised patients. Therefore, biosafety of live attenuated vaccines needs to be carefully evaluated before proceeding to clinical use.

Although a few SARS-CoV and MERS-CoV live attenuated vaccines have demonstrated efficacy in animal models, none of them have proceeded to clinical trials [114–117]. The envelope (E) protein, besides its structural roles, has a major role in inflammasome activation and is associated with exacerbated inflammation in the lung [118]. As a result, the deletion of E protein can lead to the decreased virulence of coronavirus [119]. Lamirande et al. has reported that SARS-CoV mutants lacking the E gene can induce protective effects in hamsters against SARS-CoV challenge [114]. In addition, nonstructural protein 16 (nsp16) is another viable target for the coronavirus vaccine. Nsp16 encodes ribose 2′-*O*-methyltransferase that is required for 5′ capping of viral RNA [120]. This methylation helps coronavirus avoid the activation of type I interferon-dependent innate immune response by viral RNA, and therefore nsp16 deletion attenuates virulence [120]. Both SARS-CoV and MERS-CoV nsp16 mutant vaccines have been reported to provide protection against challenge [115, 116]. Moreover, nonstructural protein 14 (nsp14), which encodes exoribonuclease (ExoN) involved in RNA proofreading during replication, is also an useful target for live attenuated coronavirus vaccine [121]. The loss of ExoN will cause a profound decrease in replication fidelity, and lead to attenuation of coronavirus pathogenesis [121]. Graham et al. has shown that ExoN deletion can reduce SARS-CoV virulence in young, aged, immunocompromised mice, and ExoN-deleted SARS-CoV vaccine can induce a protective effect against challenge in these mice [117]. In sum, all the targets mentioned above serve as potential strategies for the development of live attenuated SARS-CoV-2 vaccine.

## Recent progress on SARS-CoV-2 vaccine development

Compared with SARS and MERS, which tended to resolve spontaneously after regional outbreak, the worldwide magnitude of the COVID-19 pandemic has made development of vaccine an unprecedented urgency. This urgent need has led to many different approaches in vaccine development considerations. First of all, unconventional vaccine platforms, such as nucleic acid vaccines and viral vector vaccines, are becoming the leading players in the race of COVID-19 vaccine development due to their ability to be developed using sequence information alone [122]. These new platforms are therefore highly adaptable to emerging pathogens, and their safety profiles have already been well examined in recent influenza, Ebola and Zika outbreaks [57]. Secondly, the clinical development process of COVID-19 vaccine has been accelerated by executing trials in parallel rather than following a linear sequence of steps. For example, multiple COVID-19 vaccine candidates directly entered clinical trials before having preclinical data in animal models, and many vaccine trials have adopted an integrated phase I/II or phase II/III approach to save time [123]. Last but not least, in order to meet the massive global need of COVID-19 vaccine, vaccine developing companies, especially the front runners, are ramping up their manufacturing capacity to the scale of ~ 1 billion doses per year [124–126]. Governments from the United States and several other countries are also playing an important role in funding the scale-up of potentially effective vaccines [127–129].

In this section, we will discuss the latest preclinical and clinical development of COVID-19 vaccines (as of Oct 26, 2020). We will highlight representative COVID-19 vaccines from each major vaccine platform that have published clinical data (Table 2).

## Protein subunit vaccine

Up to now, there have been 13 SARS-CoV-2 protein subunit vaccines entering clinical trials [130]. Among these vaccines, a leading company Novavax, with its NVX-CoV2373 vaccine, has entered a phase IIb trial in South Africa (NCT04533399) and a phase III trial in the UK (2020-004123-16). NVX-CoV2373 contains a prefusion stabilized full-length spike protein adjuvanted with their proprietary saponin-based adjuvant [131, 132]. In a preclinical trial, the vaccine induced neutralizing antibodies and prevented viral replication in the respiratory tract in macaques challenged with the virus [131]. The vaccine also induced binding and neutralizing antibodies in all participants in the phase I trial [132]. In their phase I trial, they also observed a dose sparing effect by the adjuvant. They found that both adjuvanted 5 ug and 25 ug dose regimens induced significantly high titers of neutralizing antibody compared to the placebo group and the 25 ug dose without adjuvant group [132]. Another vaccine that has entered the phase II trial is Anhui Zhifei Longcom’s recombinant new coronavirus vaccine (NCT04466085). Instead of using the full-length S protein, Anhui Zhifei Longcom’s vaccine only contains the RBD of the SARS-CoV-2 S protein. However, no further design or data has been provided so far. For the other candidate SARS-CoV-2 protein subunit vaccines, most of them also utilize either full-length S protein or the RBD of S protein as their vaccine antigen. Notably, one recent study has described a generalizable strategy to enhance the immunogenicity of protein subunit coronavirus vaccines [133]. They identified a disulfide-linked dimeric form of MERS-RBD that is significantly more immunogenic and protective than its conventional monomeric counterpart [133]. They applied the same strategy to SARS-CoV-2 and has demonstrated a 10–100-fold enhancement of neutralizing antibody titers [133]. Therefore, this framework of immunogen design could be universally applicable to all protein subunit coronavirus vaccines in the future.

## DNA vaccine

There are 4 DNA vaccines for SARS-CoV-2 currently under clinical trials [130]. Among these developers, Inovio is a leading company that has published results on MERS-CoV and SARS-CoV-2 DNA vaccines. Inovio’s SARS-CoV-2 DNA vaccine INO-4800 encodes the full length S protein and is administered intradermally with a hand-held device CELLECTRA to electroporate the skin cell [70, 134]. Having experience in the phase I/IIa trial of their MERS vaccine (INO-4700), they are using the same platform for the SARS-CoV-2 vaccine INO-4800 [69, 70]. They have demonstrated that the vaccine induces neutralizing antibodies and Th1-skewed immune responses in animal models including mice, guinea pigs, and rhesus macaques [70, 135]. The vaccine is now in two phase I/II trial (NCT04447781 and NCT04336410). The interim analysis of the two phase I trials showed it induced humoral and T cell immune responses in 94% participants after two doses while only caused adverse events of grade 1 or below [136].

## RNA vaccine

Although there were no RNA vaccine studies for SARS-CoV or MERS-CoV in the past two decades, there have already been 6 novel RNA vaccines reaching clinical trials for SARS-CoV-2 since the outbreak of COVID-19 [130]. RNA vaccines consist of viral antigen-encoding messenger RNAs that can be translated by human cells to produce antigenic proteins and stimulate the immune system. RNA vaccines are usually delivered in complex with additional agents, such as protamine or lipid- and polymer-based nanoparticles, to increase its efficacy [137]. Similar to DNA vaccines, RNA vaccines have the advantages of being highly adaptable to new pathogens and being able to recapitulate the native conformation and modifications of antigenic proteins. Furthermore, compared with DNA vaccines, RNA vaccines have some additional benefits. Unlike DNA, RNA does not interact with host-cell DNA and therefore obviate the risks of genomic integration. Besides, RNA vaccines can be given through multiple routes including traditional intravenous injection, whereas DNA vaccines need to be administered via special devices like electroporation or gene gun. Nevertheless, RNA vaccines do have some drawbacks. Exogenous RNA can activate interferon-mediated antiviral immune response and lead to stalled translation and mRNA degradation, which suppress the efficacy of RNA vaccines [138]. In addition, interferon signaling is associated with inflammation and potential autoimmunity [139]. Even though there have not been severe cases of RNA vaccine-induced autoimmune diseases, it is important to carefully evaluate this potential adverse effect.

Moderna and BioNTech/Pfizer are the two leading developers for a SARS-CoV-2 RNA vaccine. Moderna's mRNA-1273 vaccine encodes a stabilized prefusion spike trimer, in which they substituted the amino acids at 986 and 987 with proline to stabilize the spike protein in its prefusion conformation [140]. The nucleotides of the mRNA were also modified not only to increase its translation and half-life but also to prevent activation of interferon-associated genes upon entering the cell [140]. The preliminary report for their phase I clinical trial showed that: (1) neutralizing antibodies were detected in all 45 patients after two doses of immunization; (2) antibody titers of immunized patients were higher than convalescent serum after two doses of vaccination; (3) Th1-biased immune responses were observed in immunized patients [140]. There were some cases of systemic adverse events after the second dose of vaccination, but no grade 4 adverse events were observed [140]. They concluded that 100 ug can induce a satisfactory immune response and thus will continue to use 100 ug dosage in phase III clinical trial (NCT04470427) [140]. In addition, they also expanded the same phase I trial to include 40 elderly participants with their age older than 55 years old [141]. Their result demonstrated that 100 ug dose of mRNA-1273 induced higher binding- and neutralizing-antibody titers than the 25 ug dose, and the adverse events associated with mRNA-1273 were mild or moderate in these elderly participants [141]. On Nov 16, 2020, Moderna revealed the first interim analysis of their phase III trial (NCT04470427) [142]. Their result showed that among 95 people who developed symptomatic COVID-19 after volunteering in this trial, only 5 of them were from the mRNA-1273 group, and the rest 90 cases were from the placebo group, resulting in a estimated vaccine efficacy of 94.5% [142]. In addition, there were 11 volunteers who developed severe COVID-19 symptoms, and their analysis showed that all 11 cases were in the placebo group and none in the mRNA-1273 group [142]. Their concurrent safety review also did not notice any significant safety concern [142]. Therefore, their promising result suggested that the mRNA-1273 vaccine is safe and effective in preventing symptomatic COVID-19. 

BioNTech and Pfizer’s mRNA vaccine has four candidates, BNT162b1, BNT162b2, BNT162a1 and BNT162c2. BNT162b1 and BNT162b2 are both nucleoside modified mRNA (modRNA) vaccine [143]. BNT162b1 encodes a trimerized RBD of spike protein while BNT162b2 encodes a full-length spike protein [143]. On the other hand, BNT162a1 is a uridine mRNA (uRNA)-based vaccine and BNT162c2 is a self-amplifying mRNA (saRNA)-based vaccine [143]. Up to now, BioNTech and Pfizer have published two BNT162b1 phase I/II trial results that were conducted in Germany (NCT04380701) and the US (NCT04368728), respectively [144, 145]. Both studies showed that the two-dose regimen of BNT162b1 elicited RBD-binding and neutralizing antibodies with titers above convalescent human serum [144, 145]. Analysis of cell-mediated immune responses showed Th1-skewed response in most participants, as demonstrated by the detection of IFNγ, IL-2 and IL-12 but not IL-4 in their assay [144, 145]. Although the German trial and the US trial used different dosages of vaccine, the two trials agreed with each other and showed that a regimen of 30–50 ug on day 1 and day 22 is able to elicit favorable immune response without severe adverse effects [144, 145]. Following these two papers, they also published another study comparing the vaccination responses between BNT162b1 and BNT162b2 [146]. BNT162b1 and BNT162b2 were shown to induce similar neutralizing titers in younger and older adults [146]. However, BNT162b2 had less systemic reactogenicity in older adults [146]. Therefore, they decided to move forward with BNT162b2 instead of BNT162b1 into a phase III clinical trial (NCT04368728). On Nov 18, 2020, Pfizer and BioNTech announced the efficacy analysis of their phase III clinical trial (NCT04368728) after meeting all primary efficacy endpoints [147]. Their evaluation showed that BNT162b2 is 95% effective against COVID-19 [147]. This result was based on analyzing 170 confirmed COVID-19 cases, of which 162 cases of COVID-19 were observed in the placebo group while 8 cases in the BNT162b2 group [147]. In addition, among 10 severe COVID-19 cases observed in this trial, 9 of them were in the placebo group and only 1 of them was in the BNT162b2 group [147]. Notably, the observed efficacy in the elderly people was over 94%, which would help protect the most vulnerable population against COVID-19 [147]. No serious safety concern was observed among 43,000 enrolled participants [147]. These data indicated BNT162b2 is another well-tolerated and efficacious COVID-19 vaccine.

## Viral vector vaccine

Currently, there are 12 viral vector vaccines in clinical trials, and an additional 36 viral vector vaccines under preclinical development [130]. Many viral vector platforms that have been tested in SARS-CoV and MERS-CoV are being explored in COVID-19 vaccines, including adenovirus (both human and non-human primates), measles virus, modified vaccinia virus Ankara (MVA), parainfluenza virus, rabies virus and vesicular stomatitis virus (VSV) [130]. Surprisingly, Venezuelan equine encephalitis (VEE) virus, which has been extensively studied in SARS and MERS vaccine, hasn’t been tested in any COVID-19 vaccine studies yet. On the other hand, influenza virus vector, which hasn’t been explored for SARS and MERS viral vector vaccines, are now gaining popularity for the development of COVID-19 viral vector vaccine [130]. For COVID-19 viral vector vaccines that have entered clinical trials, 8 out of 12 are based on adenoviruses, and the four leading candidates in this platform are AZD1222 (or ChAdOx1 nCoV-19, developed by Astrazeneca and Oxford University), Gam-COVID-Vac (or Sputnik V, or rAd26S+rAd5-S, developed by Gamaleya Research Institute), Ad5 (developed by CanSino Biological Inc. and Beijing Institute of Biotechnology), and Ad26 (developed by Johnson & Johnson and Beth Israel Deaconess Medical Center) [130].

AZD1222 is a chimpanzee adenovirus-based viral vector vaccine (ChAdOx1) expressing SARS-CoV-2 spike protein [99]. This ChAdOx1 platform has been used to develop MERS-CoV vaccine, which has demonstrated promising preclinical and phase I clinical trial data [94–98]. The AZD1222 vaccine team published their phase I/II trial interim report in July 2020 and showed that AZD1222 can elicit S protein-specific antibody and T-cell response and induce neutralizing antibody in all participants after the prime-boost regimen [99]. No severe adverse effect has been observed [99]. Based on this promising data, AZD1222 launched phase II/III trials in UK (2020-001228-32) and phase III trials in Brazil (ISRCTN89951424), United States (NCT04516746), Russia (NCT04540393) and India (CTRI/2020/08/027170). In Sep 2020, the AZD1222 phase II/III trial in the UK was once put on hold for safety review because a participant has developed unexplained illness, but following later independent review in the UK determined that the trial is still safe and therefore the AZD1222 clinical trial resumed [148, 149]. On Nov 23, 2020, Astrazeneca annouced the interim analysis of their clinical trial in UK (2020-001228-32) and Brazil (ISRCTN89951424) [150]. Their pooled result showed that AZD1222 has an average efficacy of 70%, based on analyzing a total of 131 COVID-19 cases from 11,636 volunteers [150]. Interestingly, one dose regimen showed 90% efficacy when AZD1222 was given as half first dose followed by a full second dose (*n* = 2,741) [150]. On the other hand, two full dose regimen had only 62% efficacy (*n* = 8,895) [150]. Due to the response discrepancy between different subgroups, additional trials may be needed to better determine the efficacy and the most suitable regimen of AZD1222. In addition, the Gam-COVID-Vac vaccine team has published their phase I/II trial results [151]. They conducted two different trials, with one using frozen formulation (NCT04436471) and the other using lyophilized formulation (NCT04437875) of the vaccine [151]. In both phase II trials, they tested their patients with heterologous prime-boost immunization of recombinant adenovirus type 26 vector encoding SARS-CoV-2 spike glycoprotein (rAd26-S) plus recombinant adenovirus type 5 vector encoding SARS-CoV-2 spike glycoprotein (rAd5-S) [151]. Their results showed that both frozen and lyophilized formulation of the vaccine induced potent neutralizing antibodies and CD4+ and CD8+ T-cell immune responses, with the immune response of frozen formulation being slightly stronger than the lyophilized formulation [151]. Both vaccines were safe and well-tolerated in all participants [151]. Now this vaccine is also entering phase III trial in Russia (NCT04530396) and Belarus (NCT04564716). On Nov 24, 2020, Gamaleya Research Institute announced the second interim analysis of Gam-COVID-Vac (or Sputnik V) phase III clinical trial (NCT04530396) [152]. Their result showed that Gam-COVID-Vac had a efficacy of 91.4% on Day 28 after the first dose, which was based on analyzing 39 confirmed cases among 18,794 volunteers [152]. They also revealed that on Day 42 after the first dose (Day 21 after the second dose), the vaccine efficacy was even above 95% [152]. There were no unexpected adverse effect documented during the trial [152]. These promising results suggested that Gam-COVID-Vac is safe and effective in preventing COVID-19. Furthermore, the Ad5 vaccine team, whose vaccine is based on human adenovirus 5, has also published their clinical data [92, 93]. In their phase II study, Ad5-vectored COVID-19 vaccine induces significant neutralizing antibodies and T-cell mediated immune response after single immunization [93]. They tested two dosage, 1 × 10^11^ and 5 × 10^10^ viral particles, and showed that the 5 × 10^10^ dose causes less severe adverse reactions without compromising the immunogenicity [93]. Now this vaccine has advance to two phase III global multi-centered clinical trials (NCT04526990 and NCT04540419). Finally, Johnson & Johnson’s Ad26-based COVID-19 vaccine has also entered phase III clinical trial (NCT04505722), but no data from its earlier trial has been reported yet.

## Whole inactivated vaccine

Currently, there are 7 whole inactivated COVID-19 vaccine in clinical trials [130]. From the previous experience of SARS-CoV and MERS-CoV vaccine development, whole inactivated virus can induce adverse effect such as eosinophil-related lung immunopathology in preclinical models [36, 37]. Even though no serious adverse effect has been reported for whole-inactivated COVID-19 vaccine, it is important for the research community to keep this in mind and carefully evaluate potential adverse effects. For all ongoing trials of whole inactivated COVID-19 vaccine, three of them have publicly reported their preclinical or clinical data. SinoVac Inc. developed CoronaVac (also known as PiCoVacc), which is a beta-propiolactone inactivated, Vero cell line propagated whole virus vaccine originated from a patient-derived CN-2 SARS-CoV-2 virus strain [153]. In their preclinical study, PiCoVacc induces broad neutralizing antibodies against 10 representative SARS-CoV-2 strains in mice, rats, and non-human primates [153]. Immunizing macaques with three doses of PiCoVacc provides them with protective immunity against SARS-CoV-2 challenge without causing any antibody-dependent enhancement effect [153]. Following their preclinical study, CoronaVac has completed two phase I/II trials (NCT04383574 and NCT04352608, result not yet published) and is now starting phase III clinical trial in Brazil (NCT04456595), Indonesia (669/UN6.KEP/EC/2020) and Turkey (NCT04582344). In addition, Sinopharm Inc. and Wuhan Institute of Biological Products have developed a different COVID-19 inactivated virus vaccine (no specific product name). In this vaccine, WIV04 strain was isolated from a COVID-19 patient in Wuhan, propagated in Vero cells, and followed by two rounds of beta-propiolactone inactivation [154]. They tested three different dosage and three different injection timelines in their phase I and phase II studies, and their phase I/II interim report showed that patients receiving different vaccination regimen all had demonstrated neutralizing antibodies and with only a low rate of adverse reactions [154]. Now they have launched a phase III clinical trial in United Arab Emirates (ChiCTR2000034780) and Kuwait (ChiCTR2000039000). Finally, Sinopharm Inc. also collaborated with Beijing Institute of Biological Products to develop another COVID-19 inactivated virus vaccine BBIBP-CorV [155, 156]. The manufacturing process of BBIBP-CorV is very similar to the other vaccine Sinopharm Inc. produced, except that BBIBP-CorV used a different HB02 strain rather than WIV04 strain [155]. They have tested BBIBP-CorV in preclinical models and showed that two-dose immunization of BBIBP-CorV can protect rhesus macaques from SARS-CoV-2 challenge [155]. Following this, they completed a clinical phase I/II trial with BBIBP-CorV and demonstrated that BBIBP-CorV is safe and well-tolerated in all tested doses in two age groups [156]. Furthermore, their immunogenicity result showed that humoral responses against SARS-CoV-2 were induced in all vaccine recipients 42 days after immunization [156]. Now this vaccine is under phase III clinical trial in United Arab Emirates (ChiCTR2000034780) and Argentina (NCT04560881).

## Other vaccine platforms

There are also several other COVID-19 vaccine candidates using different technologies other than the platforms mentioned above. Virus-like particle-based vaccine, which has been demonstrated to induce humoral and cell-mediated immunity in SARS-CoV and MERS-CoV preclinical models, has one candidate COVID-19 vaccine in phase I clinical trial (NCT04450004) and 14 vaccine candidates under preclinical development [130]. However, none of the group has publicly reported their vaccine studies yet. Live attenuated vaccine, which has been shown to provide protective effect in SARS-CoV and MERS-CoV challenged mice, has 3 preclinical ongoing studies [130]. The higher risk of adverse effect has made live attenuated vaccine a less appealing choice for the time-sensitive race of COVID-19 vaccine development. Nevertheless, if successfully developed, live attenuated vaccine can provide the most potent protective effect due to its high similarity to natural infection.

In addition to these traditional platforms, scientists have also developed COVID-19 vaccines using unconventional approaches. Aivita Biomedical, Inc. has developed AV-COVID-19, which is an autologous dendritic cells vaccine loaded with SARS-CoV-2 antigens [157]. AV-COVID-19 is derived from patients’ own peripheral blood monocytes, then differentiated in vitro into dendritic cells, and incubated with SARS-CoV-2 antigens before injecting back into patients’ blood [157]. Now the company has launched a phase I/II clinical trial to evaluate its safety and efficacy profile in adults (NCT04386252). Besides, Symvivo Corporation has developed bacTRL-Spike, a live Bifidobacterium vaccine engineered to deliver synthetic plasmid DNA encoding spike protein from SARS-CoV-2. They have also registered a phase I clinical trial to examine the safety of this vaccine (NCT04334980). Moreover, a group from Nanjing University has found that a plant microRNA, MIR2911, can target SARS-CoV-2 by binding to their mRNA and blocking protein translation [158]. Their data showed that MIR2911 inhibited SARS-CoV-2 replication and accelerated negative conversion of infected patients [158]. Following their study, they are now launching a phase I clinical trial (ChiCTR2000031432) in China to evaluate the safety and tolerance of MIR2911 in patients.

Finally, people have been testing existing licensed vaccines and trying to repurpose them to combat COVID-19. It has been shown that tuberculosis vaccine bacillus Calmette–Guérin (BCG) can train innate immunity and induce nonspecific host defensive reaction against viral pathogens, including respiratory syncytial virus (RSV), influenza A virus and herpes simplex virus type 2 (HSV2) [159–162]. Additionally, an interesting study compared the national difference in COVID-19 impact and correlated it with national BCG vaccination policy [163]. They found that countries without universal policies of BCG vaccination have been more severely affected compared to countries with universal and long-standing BCG policies [163]. Based on these rationales, there have been at least 13 phase III clinical trials testing whether BCG vaccine can reduce the morbidity and mortality of healthcare workers (NCT04328441, NCT04327206, NCT04350931, NCT04348370, NCT04362124, NCT04369794, NCT04373291, NCT04379336, NCT04384549, NCT04439045, NCT04387409, NCT04417335, NCT04414267).

## Additional considerations for SARS-CoV-2 vaccine development

Given the rapid transmission and asymptomatic spread of COVID-19, it is clear that an effective vaccine with global immunization coverage is required to bring people’s lives back to normalcy. However, even when an effective SARS-CoV-2 vaccine becomes available, the duration of vaccine-induced immunity is still largely unknown. Previous SARS studies have shown that SARS-specific IgG and neutralizing antibodies were only maintained for approximately 2 years in patients who recovered from SARS-CoV infection [164, 165]. As a result, permanent immunity is less likely to be the case for COVID-19 vaccines, and a regular vaccination policy might be required in the future. In addition, it is still unclear what is the minimal neutralizing antibody titer that can provide protective effect against SARS-CoV-2 infection. It is believed that the higher neutralizing antibody vaccination induces, the better protective effect it will be. This is consistent with the observation that most COVID-19 reinfection cases only experience mild or no symptom during their first infection, which might not be sufficient to induce strong neutralizing antibodies [166, 167]. Therefore, it is of great importance that further studies characterize the correlation between neutralizing antibody and protective effect to guide COVID-19 vaccine development. Last but not least, various mutations have been detected in the SARS-CoV-2 genome, with D614G mutation being the most prevalent one [168]. D614G is a missense point mutation in S protein that increases the infectivity of SARS-CoV-2 by decreasing S1 shedding and increasing S protein incorporation into virion [169, 170]. Fortunately, D614G mutation does not prevent neutralizing antibodies from binding to SARS-CoV-2 and thus does not provide resistance to vaccination [170]. However, it is possible that such immune-escaping mutations appears in the future and makes COVID-19 vaccine development even more difficult.

## Concluding remarks

Since the discovery of human coronaviruses in 1960s, new types of coronaviruses have kept emerging and have gradually become a serious threat to global public health. Even though there have been almost two decades since the first coronavirus outbreak, the scientific and medical community are not well prepared with effective weapons to combat these pathogens. One lesson we learned from this is that the financial and regulatory mechanism of current pharmaceutical market does not provide enough incentive to encourage vaccine development before a deadly outbreak happens. To make up for this, now academic institutions and companies all over the world are developing an explosive numbers of vaccine candidates with highly compressed clinical trial schedules. Fortunately, the biological and clinical lessons we learned from SARS-CoV and MERS-CoV researches, together with the vaccine development experience we gained from other diseases, have already guided us to come up with multiple promising candidate solutions. Besides, multiple therapeutic candidates targeting molecules in SARS-CoV-2 life cycle and human immune response against COVID-19 have also been rapidly explored, with Remdesivir and Dexamethasone being the two leading drugs that showed promising clinical evidences in shortening the time to recovery and decreasing mortality rates [171, 172]. These treatment options can be complementary to SARS-CoV-2 vaccines to achieve overall mitigation of the COVID-19 pandemic. In conclusion, we hope countries all over the world, regardless of political ideologies, can unite and work together to achieve fast and successful COVID-19 vaccine development in the near future.

## Data Availability

Not applicable.

## Abbreviations

COVID-19: Coronavirus Disease 2019
CoV: Coronavirus
SARS-CoV: Severe Acute Respiratory Syndrome Coronavirus
MERS-CoV: Middle East Respiratory Syndrome Coronavirus
SARS-CoV-2: Severe Acute Respiratory Syndrome Coronavirus 2
pp: Polyproteins
Nsps: Non-structural proteins
S: Spike
E: Envelope
M: Membrane
N: Necleocapsid
RBD: Receptor binding domain
ACE2: Angiotensin-converting enzyme 2
DPP4: Dipeptidyl peptidase-4
ER: Endoplasmic reticulum
ADE: Antibody-dependent enhancement
NTD: N-terminal domain
IM: Intramuscular
SC: Subcutaneous
VLP: Virus-like particle
CPV: Canine parvovirus
VEE: Venezuelan equine encephalitis
VRP: Virus replicon particle
Ad#: Human adenovirus type #
ChAdOx1: Chimpanzee Adenoviral Vector
ExoN: Exoribonuclease
modRNA: Modified RNA
uRNA: Uridine mRNA
saRNA: Self-amplifying RNA
RSV: Respiratory synctial virus
HSV: Herpes simplex virus
